# Evolutionary Responses to Invasion: Cane Toad Sympatric Fish Show Enhanced Avoidance Learning

**DOI:** 10.1371/journal.pone.0054909

**Published:** 2013-01-23

**Authors:** Georgina Caller, Culum Brown

**Affiliations:** Department of Biological Sciences, Macquarie University, Sydney, Australia; University of Pretoria, South Africa

## Abstract

The introduced cane toad (*Bufo marinus*) poses a major threat to biodiversity due to its lifelong toxicity. Several terrestrial native Australian vertebrates are adapting to the cane toad’s presence and lab trials have demonstrated that repeated exposure to *B. marinus* can result in learnt avoidance behaviour. Here we investigated whether aversion learning is occurring in aquatic ecosystems by comparing cane toad naïve and sympatric populations of crimson spotted rainbow fish (*Melanotaenia duboulayi*). The first experiment indicated that fish from the sympatric population had pre-existing aversion to attacking cane toad tadpoles but also showed reduced attacks on native tadpoles. The second experiment revealed that fish from both naïve and sympatric populations learned to avoid cane toad tadpoles following repeated, direct exposure. Allopatric fish also developed a general aversion to tadpoles. The aversion learning abilities of both groups was examined using an experiment involving novel distasteful prey items. While both populations developed a general avoidance of edible pellets in the presence of distasteful pellets, only the sympatric population significantly reduced the number of attacks on the novel distasteful prey item. These results indicate that experience with toxic prey items over multiple generations can enhance avoidance leaning capabilities via natural selection.

## Introduction

The human facilitated introduction of novel organisms into new ecosystems has been occurring, both intentionally and accidentally, for thousands of years [1], [2]. The vast majority of these species die within a few generations and of those that successfully persist most will have little impact on their new environment [3]. However, a small proportion of persisting introduced species, collectively known as invasive species, will become highly destructive, often experiencing rapid, uncontrollable population growth and come to dominate the habitats they occupy [2], [4]. Invasive species represent a key threat to biodiversity, with an estimated 40% of modern extinctions at least partially caused by invasive species [5].

Whilst studying the responses of native species to invasion has obvious conservation applications, it also offers a unique opportunity to study and critically examine evolutionary processes [6]. Invasive species present native organisms with a novel threat, as they are more likely to come from clades that are not present in the native ecosystem [7]. While many species succumb to extinction, there are numerous accounts of local species adapting to the presence of invasive species via phenotypic plasticity or natural selection [6], [8], [9]. Evolutionary adaptation develops over several generations but is inflexible on an individual temporal scale and is thus relatively slow to respond to novel threats [10]. On the other hand phenotypic plasticity is relatively rapid, allowing animals to adapt to environmental change with little loss of genetic variation [11], [12]. While some of this plasticity is morphological, it most often occurs through the learning process as a result of interactions with the invasive species [13], [14]. In this way the learning ability of animals is a key aspect to overcoming the threat posed by invasive species and other rapid ecosystem shifts [15].

The cane toad (*Bufo marinus*) was introduced to Queensland in 1935 in an attempt to control sugar cane pests, and has since been spreading uncontrollably across Australia [16], [17], [18]. The cane toad is set apart from the other invasive species of Australia by its toxicity. The toad’s body contains a myriad of poisonous chemicals that exist in various combinations throughout its lifecycle, making it a major threat to predators in both aquatic and terrestrial ecosystems [19], [20], [21], [22]. Entrance of the cane toad into an area has correlated with large drops in population size of numerous terrestrial predators, such as dingoes, snakes and lizards [23], [24], [25], [26]. Furthermore, lab trials have confirmed that cane toad eggs and tadpoles are lethal to numerous aquatic predators, including invertebrates, fish, amphibians and reptiles [19], [27], [28].

Recent studies have found evidence of native predators adapting to cane toad presence. Black snakes (*Pseudechis porphyriacus*) have developed a heritable behavioural aversion and toxin resistance to cane toads [29]. Many cane toad-predating snakes have evolved a smaller head, preventing them from ingesting toads large enough to deliver a lethal dose of toxin [30]. Furthermore, there is evidence that black kites (*Milvus migrans*) have learnt to flip cane toads onto their backs to consume the innards, thus avoiding the poison producing paratoid glands [31]. Laboratory trials have succeeded in eliciting adaptive learning responses to cane toads from aquatic predators. The marbled frog (*Limnodynastes convexiusculu*) ceased attempting to consume cane toads and ignored them as a possible food source after repeated direct exposure to live cane toad metamorphs [32]. Similar responses to cane toad tadpoles have been observed in other aquatic predators; northern trout gudgeons (*Mogurnda mogurnda*), Dahl's aquatic frog (*Litoria dahlii)*, sooty grunter (*Hephaestus fuliginosus*) and barramundi (*Lates calcarifer*) ceased attacking cane toad tadpoles after repeated exposure [33], [34]. Thus far, however, no studies have investigated whether the learnt aversion to cane toad tadpoles occurs in the wild or whether the presence of toxic prey items has evolutionary consequences for avoidance learning in general.

Here we determined if aquatic predators are adapting to the presence of cane toads by addressing the following questions: 1) Have toad sympatric fish developed an aversion to toad tadpoles that is lacking in toad naïve fish? 2) Is the aversion to toxic cane toad tadpoles a learnt behaviour? 3) Has long-term coexistence with cane toad tadpoles produced differences in general aversion learning between toad naïve and toad sympatric fish via natural selection?

## Methods

### Animal Collection and Husbandry

Naïve crimson-spotted rainbowfish *M. duboulayi* were collected in March 2010, from the Orara River in Coffs Harbour, New South Wales (153° 00.42 E, 30° 15.26S) using standard bait traps. Cane toads had not entered the area at the time of fish collection. Toad sympatric *M. duboulayi* were collected from Juff’s Crossing on the Pine River, near Dayboro, Queensland (152° 48.486E, 27° 12.378S) in March 2010 and from JC Slaughter Falls Park dam (152° 57.847E, 27° 28.567S) in April 2010, using standard bait traps and Seine nets. The populations of *M. duboulayi* from these areas have coexisted with cane toads for approximately 60 years (Sabath *et al.* 1981). The fish from each location did not differ significantly in size (alloparic fish mean 4.5±0.8 cm; sympatric fish 4.4±0.8 cm). Native tadpoles (*Litoria wilcoxi*) were also collected from Juff’s Crossing using hand held dip nets. This species of tadpole is common along the east coast of Australia and was present at all collection sites. It’s distribution completely overlaps with the distribution of *M. duboulayi* and is a high quality prey item for this species. This species is a deep mottled brown colour in comparison to the midnight back of the canetoad tadpole. *B. marinus* tadpoles of a similar size to the native tadpoles were collected from an unnamed creek in Dayboro, Queensland (152° 49. 459E, 27° 12. 030S) in March 2010 and Darwin Botanic Gardens (130°50.11E, 12° 26.42S) using small hand-held dip nets.

Fish were maintained in groups of 20 in 90 cm×34 cm×50 cm tanks filled with aged, dechlorinated water, with a standard temperature of 26 C, a cycle of 12 hours light and 12 hours dark, and fed commercial flake food. Fish were left for two weeks to acclimatise to captivity before testing began. As *M. duboulayi* can retain learning based on negative experiences for at least 11 months [35], we were confident that an acclimatisation period of this length would not have any significant effect on the fishes’ reaction to toad tadpoles.

Tadpoles were kept in several 37 cm×21 cm×29 cm tanks of aged, dechlorinated water, oxygenated with air stones. They were fed frozen lettuce and algae every 1–2 days, with small water changes after each feeding to maintain water quality.

### Experiment 1 Procedure: Recognition Test

This procedure was designed to test any pre-existing biases to attack native or cane toad tadpoles. Sympatric (*n* = 26) and naïve (*n* = 24) fish were moved into individual 60 cm×30 cm×60 cm (water level 30 cm) experimental tanks and allowed 24 hours to acclimatise, fish were not fed during this period in order to enhance their feeding motivation. Experimental tanks were maintained with the same conditions as the housing tanks, but lacked gravel or any furnishings to make the tadpoles more conspicuous. A 24 cm×20 cm ‘vial zone’ was marked at both ends of the testing tanks, with the rest of the tank considered a ‘neutral zone’. Both filter and heater were placed in the neutral zone so as not to confound the results. Vials were 4.3 cm×7.5 cm cylinders of perforated (hole 2 mm diameter) transparent plastic, allowing fish to both see and detect any chemical cue given off by the tadpoles without directly interacting with them.

All fish were exposed to two treatments administered in random order: Toad Only (one vial with four *B. marinus* tadpoles, one empty vial) and Native Only (one vial with four *L. wilcoxi* tadpoles, one empty vial). Treatments lasted for ten minutes each, with a rest period of one hour in between. Fish interest was measured by three behaviours; approach; fish faced the vial and swam towards it (if the fish was outside the ‘vial zone’ then the approach was only recorded if it entered the vial zone during the approach). Attack; fish pecked at the vial, making physical contact, and Time in proximity; the amount of time fish spent in each vial zone. After experimentation, fish were fed to confirm feeding motivation; fish that did not feed after experimentation were excluded from the data set (n = 1 sympatric fish).

### Experiment 2 Procedure: Direct Exposure

This procedure determined if fish reduced their attacks on cane toad tadpoles after repeated, direct exposure and if this negative experience was generalised to a reduction in native tadpole consumption. Fish were introduced to individual testing tanks following the procedure from Experiment 1. Testing tanks were 45 cm×35 cm×60 cm (water level 22 cm), but were otherwise identical to testing tanks in Experiment 1. Fish were randomly assigned to the Toad Exposure treatment (sympatric *n* = 11, naïve *n = *10) or the Control treatment (sympatric *n* = 10, naïve *n* = 10). During the Toad Exposure treatment a single *B. marinus* tadpole was introduced to each testing tank for 10 minutes per day for five consecutive days. After the fifth toad exposure, the fish rested for one hour before the introduction of a native tadpole for 10 minutes. All fish in the Toad Exposure treatment were fed following each trial to confirm feeding motivation and avoid increasing hunger confounding the results. Fish in the Control treatment received a 10 minute native tadpole trial without the previous five days of toad tadpole exposure but were otherwise maintained in the same manner. Fish response was measured by the number of investigations (fish swam directly towards the tadpole but stopped within close proximity and did not attack), attacks (fish pecked at the tadpole or engulfed it in its mouth) and whether the tadpole was consumed.

### Experiment 3 Procedure: Novel Avoidance Task

Experiment 3 was designed to determine whether living in sympatry with cane toads has favoured the evolution of aversion learning in *M. duboulayi*. *TropiGro freshwater fish food* pellets were soaked in red or green food dye for 15 minutes. Analysis of *Melanotaenia* retinas indicate they should be able to distinguish between these two colours [48], (J. Kelley pers comm). Distasteful pellets (DP) were soaked overnight in a quinine solution (10^−3^ moles per 500 g water). Quinine is universally distasteful to fish and, like cane toad tadpoles, is bitter tasting [36], [37]. Edible pellets (EP) were soaked overnight in water. Twenty-four fish (12 from each population) were weened onto undyed pellets in the week preceding the experiment.

Fish were introduced to 33 cm×36 cm×39 cm (water level 23 cm) testing tanks following the procedure of Experiment 1. Fish first underwent five control trials, in which they were presented with either a red or a green EP for two minutes, followed by an EP of the other colour for two minutes, in order to confirm that the fish were consuming dyed pellets and to determine if there was any colour bias. The control trials were administered over a single day and fish were required to eat both pellets in all trials in order to proceed to the aversion trials. Only one fish from the naïve population failed this test.

Each fish was randomly assigned either the red or green pellet as the DP. A learning trial consisted of introducing either the DP or the EP into the testing tank for two minutes, followed by introduction of the other pellet for two minutes. Any uneaten pellets were removed after two minutes. The order of pellet introduction was randomly assigned each trial. Each fish received ten trials per day over five consecutive days.

Interest in the pellets was measured by three behaviours: investigative approach (fish swam directly towards the pellet but stopped within close proximity and did not attack), attack (fish pecked at the pellet or engulfed it in its mouth) and whether the pellet was consumed (only recorded if the entire pellet was eaten). If the fish did not eat any pellets for five trials in a row, then the trials for that fish were stopped for the day. If the fish did not eat any pellets for the first three trials of the following day we concluded that it developed an aversion to feeding on pellets and the fish was retired from the experiment.

### Statistical Analysis

For experiment 1, we analysed both the raw data and the proportion of all attacks/approaches aimed at tadpoles. A repeated measure analysis of variance (ANOVA) was used to assess the difference between populations with respect to their interest in the tadpoles. For experiment 2, population differences in the Control treatment and Native trial were analysed using an ANOVA. The data from the five exposures to the cane toad tadpoles was analysed using a repeated measures ANOVA as were the data for Experiment 3. Both of these data sets were log (+2) transformed prior to analysis.

### Ethical Note

All experiments conducted herein were authorised by the Macquarie University Ethics Committee under ARA 2010/014.

## Results

For simplicity we only present the data on the number of attacks, unless otherwise stated, since all metrics showed qualitatively similar results. Fish size and treatment order had no statistically significant effects.

### Experiment 1: Recognition Test

Naïve allopatric fish made significantly greater numbers of attacks on both cane toad tadpoles (ANOVA: F_1_,_47_ = 10.539, P = 0.002) and native tadpoles (ANOVA: F_1,47_ = 13.992, P = 0.0005) than sympatric fish. However the two populations did not differ in the number of attacks made against empty vials (ANOVA: toad only treatment F_1,47_ = 1.537, P = 0.2211, native only treatment F_1,47_ = 0.982, P = 0.327).

**Figure 1:**
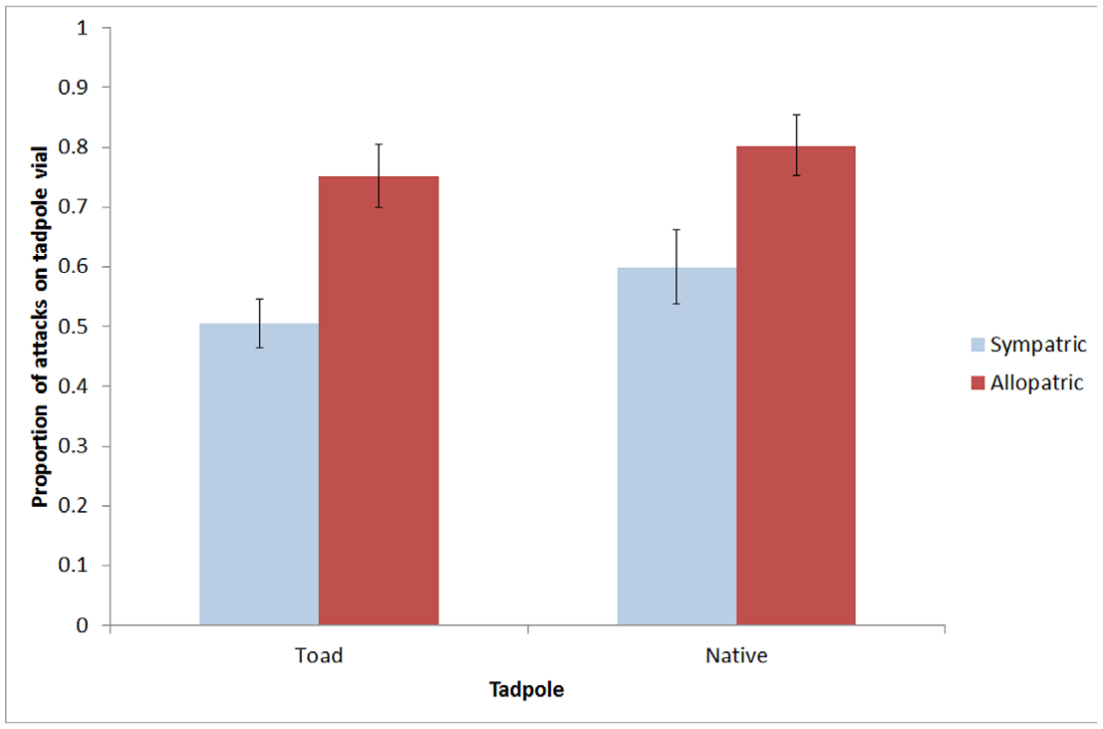
The the mean proportion of attacks (±SE) on the vial containing tadpoles versus an empty vial for toad sympatric and allopatric fish populations. Proportions >0.5 represent a prefernce for the vial containing the tadpoles.

Of all attacks during the Toad Only treatment, the proportion of naïve allopatric fish attacks on the toad tadpoles was significantly greater than the proportion of sympatric fish attacks on the toad tadpoles (ANOVA: F_1,19_ = 7.23, P = 0.015). Naïve allopatric fish showed an obvious preference for the vial containing toad tadpoles over the empty vial while sympatric fish attacked the vial containing toad tadpoles and the empty vial in almost equal proportions (Fig. 1). In the Native Only treatment, the proportion of sympatric fish attacks on the native tadpoles did not differ significantly from the proportion of naïve allopatric fish attacks on the native tadpoles (ANOVA: F_1,26_ = 2.79, P = 0.107) and both groups showed a clear preference for the vial containing native tadpoles over the empty vial (Fig. 1).

### Experiment 2: Direct Exposure

**Figure 2:**
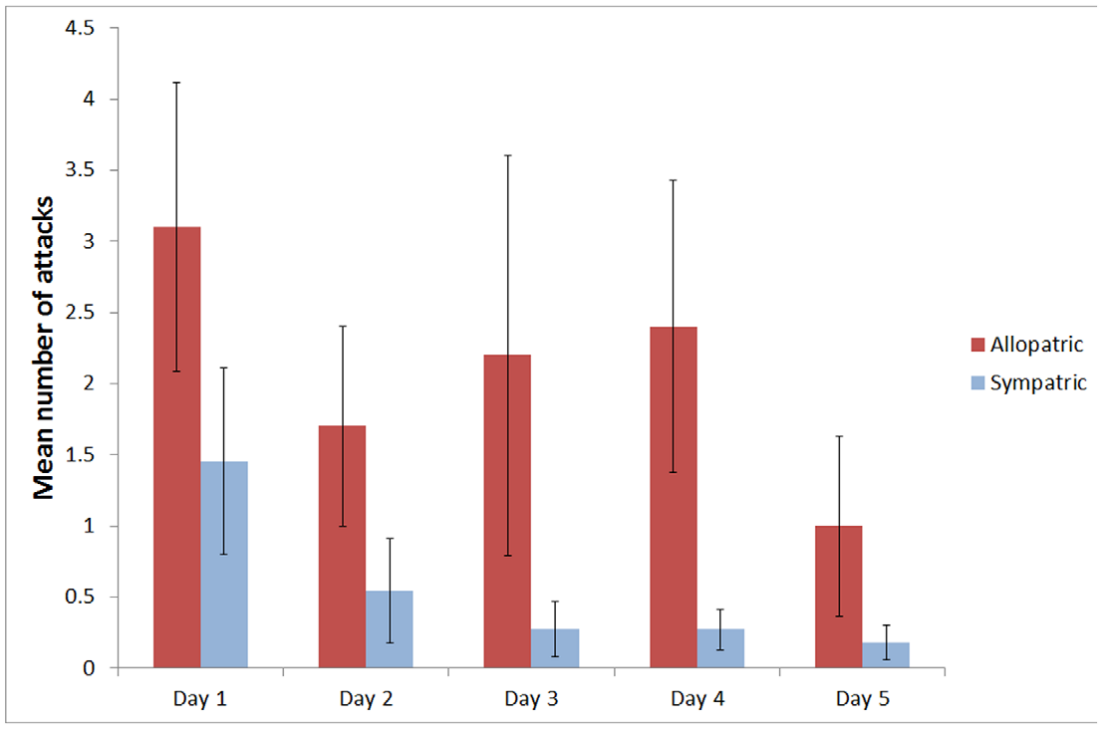
Mean number of attacks (±SE) on cane toad tadpoles over five days of direct exposure by cane toad allopatric and sympatric fish populations.

As with the first experiment, naïve fish from allopatric populations showed a greater general interest in tadpoles by attacking significantly more than sympatric fish in the Toad Exposure treatment (attack RM ANOVA: F_1,19_ = 11.193, P = 0.003; Fig. 2). The number of attacks on cane toad tadpoles for both populations tended to vary over the number of exposures (rmANOVA: F4, 76 = 2.195, P = 0.077; Fig. 2). There was no significant time by population interaction as both groups displayed a similar behaviour pattern over the five day period, despite higher attacks and approaches by naïve allopatric fish (rmANOVA: F4, 76 = 0.304, P = 0.874; Fig. 2). Post-hoc analysis revealed that there was a marginal difference in the number of attacks between treatments 1 and 5 for the allopatric fish (Mann-Whitney U: Z = 1.822, P = 0.068) but not for the sympatric fish (Mann-Whitney U: Z = 1.361, P = 0.103).

We compared the number of attacks on native tadpoles between the two populations in the control treatment (no exposure to cane toad tadpoles) and the test treatment (five exposures to cane toad tadpoles). In the control fish, naïve allopatric fish attacked native tadpoles significantly more often than sympatric fish (F_1,18_ = 5.692, P = 0.028). In the test fish, the number of attacks on native tadpoles did not differ significantly between the two populations (attack ANOVA: F_1,19_ = 2.960, P = 0.102). A comparison with the control fish revealed that this was largely generated by a decrease in the number of attacks made by allopatric fish following repeated exposure to cane toad tadpoles.

### (c) Experiment 3: Novel Avoidance Task

Fish displayed no significant colour bias, difference between populations or change over time during the control trials (P>0.5 in all cases).

**Figure 3:**
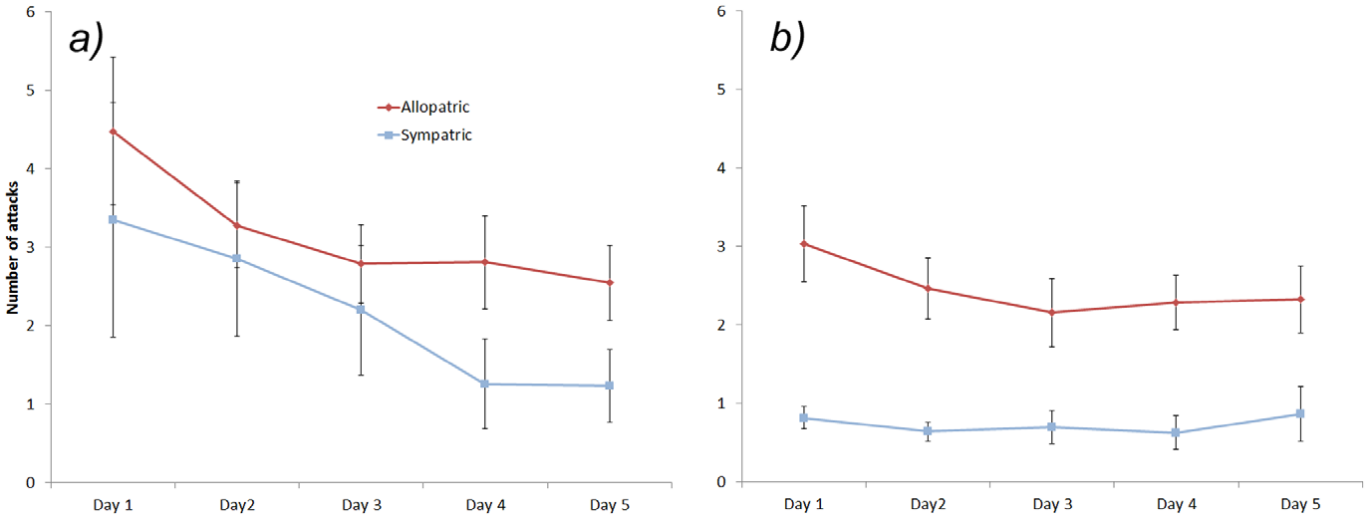
The mean (±SE) number of attacks on distasteful pellets (A) and edible pellets (B) by rainbowfish from cane toad allopatric and sympatric populations over five days.

Over all learning trials, naïve allopatric fish attacked pellets significantly more than sympatric fish (RM ANOVA: F_1,28_ = 10.539, P = 0.003). Both populations attacked DP more often than EP (RM ANOVA: F_1,28_ = 4.298, P = 0.047). The number of attacks decreased over the 5 days (RM ANOVA; F_4,112_ = 4.884, P = 0.001). A significant three-way interaction between pellet type, colour and population was also found (F_4,112_ = 2.466, P = 0.049). In order to examine the complex interaction, we split the data by treatment. The number of attacks on the distasteful pellets decreased over time (RM ANOVA; F_4,64_ = 5.181, P = 0.001; Fig. 3a) whereas the number of attacks on edible pellets did not (RM ANOVA; F_4,64_ = 5.181, P = 0.001; Fig. 3b). Closer examination of the data for distasteful pellets showed this decrease was not significant for the naïve population (RM ANOVA; F_4,44_ = 2.352, P = 0.069) but it was for the sympatric population (RM ANOVA; F_4,20_ = 4.073, P = 0.014; Fig. 3a).

**Figure 4:**
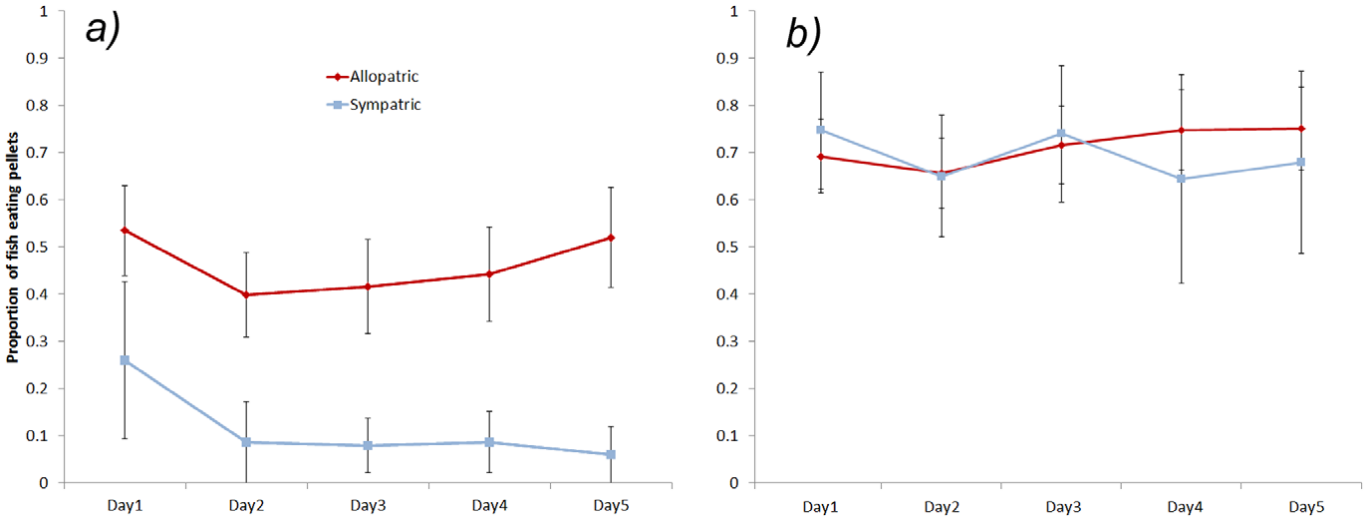
The mean (±SE) proportion of rainbowfish eating distasteful pellets (A) and edible pellets (B) from cane toad allopatric and sympatric populations over five days.

**Figure 5:**
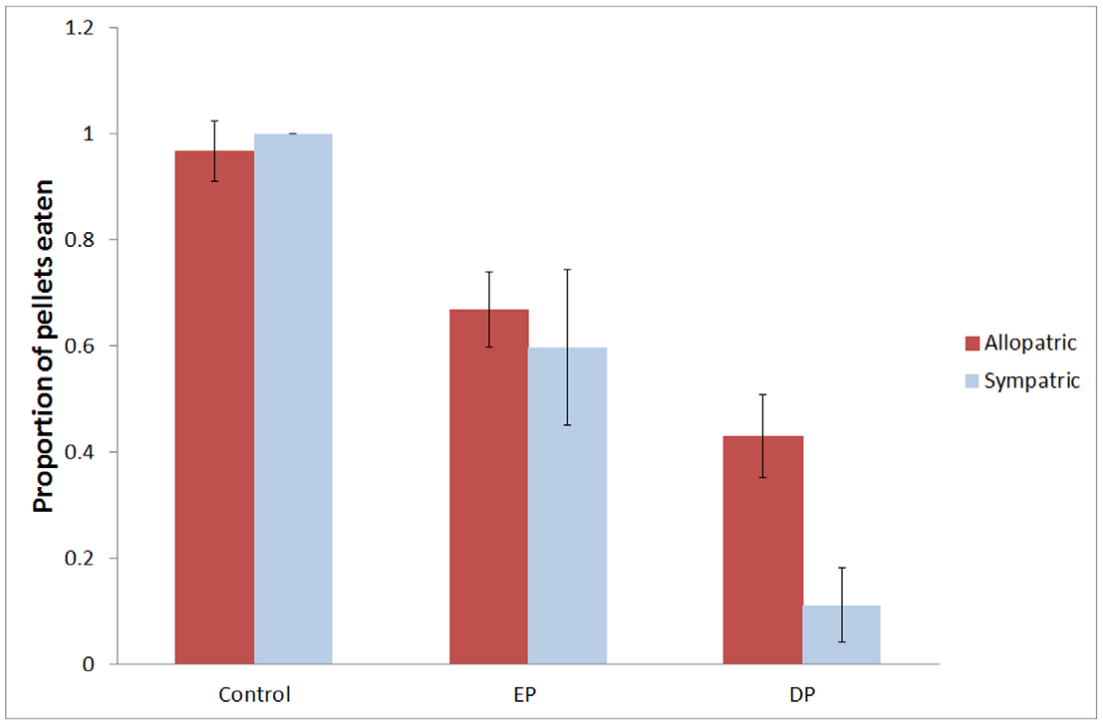
The mean proportion (± SE) of pellets eaten during the contol period when both colours were edible and during the test treatment when one colour was edible (EP) and the other was distasteful (DP).

Analysis of the likelihood that fish would eat the pellets revealed no effect of pellet colour so it was removed from the analysis. Sympatric fish ate significantly fewer distasteful pellets than naïve fish (F_1, 16_ = 5.539, P = 0.032). Sympatric fish showed a significant decrease in the number of distasteful pellets eaten after trail 1 (F_4, 20_ = 4.322, P = 0.011), while no decrease occurred in naïve fish (F_4, 44_ = 0.936, P = 0.452; Fig. 4a). No differences between populations was observed when examining the response to the edible pellet (F_1, 16_ = 0.065, P = 0.803; Fig. 4b) nor was there a differences between the populations during the control trails (ANOVA: F_1,34_ = 1.417, P = 0.242) (Fig. 5).

We then compared the proportion of pellets eaten during the experiment with the control fish that had not been exposed to DPs and found that both populations were significantly less likely to consume the DP having been repeatedly exposed to them than fish in the control group (RM ANOVA: F_1, 16_ = 144.188, P<0.001), but were also significantly less likely to consume EP (RM ANOVA: F_1, 16_ = 27. 768, P<0.001) indicating the development of a general aversion of pellets over the course of the experiment (Fig. 5).

## Discussion

Cane toad sympatric fish were less inclined to attack any kind of tadpole but were particularly less likely to attack cane toad tadpoles than were the cane toad naïve fish. This suggests that sympatric fish can recognise cane toad tadpoles without directly interacting with them even after months of life in captivity. Moreover, they evidently generalise their aversion of cane toad tadpoles to native tadpoles. Cane toad naïve fish showed no such aversion and attacked both cane toad and native tadpoles with equal vigour. When allowed to interact directly with the cane toad tadpoles, sympatric fish attacked less often than naïve fish and decreased their attacks over time. By day three they had reached the lower limit of attacks and could improve no further (Fig. 3). At this stage, on average, sympatric fish attacked just once every four trials whereas naïve fish attacked twice per trial. Nevertheless, naïve fish also eventually reduced the number of attacks despite the high level of individual variation. Importantly, prior to direct exposure there were clear differences in the number of attacks on cane toad tadpoles between the two populations, however, following direct exposure the difference was eliminated. More broadly, there is evidence that crimson-spotted rainbowfish showed avoidance learning with repeated exposure to toxic prey items which is in line with previous studies in a variety of terrestrial and aquatic taxa [32], [33], [34].

In the final experiment we produced an abstract version of the tadpole foraging test by introducing toxic prey items that were novel to both populations, thereby illuminating any biases developed through previous exposure in the wild. The results of this experiment mirrored the previous one. Naïve fish attacked pellets more often than sympatric fish and the attacks on edible pellets did not decline over time but the attacks on distasteful pellets did (Fig. 3). Importantly the decline in attacks was only significant in the cane toad sympatric population. These data on the number of attacks were mirrored in the likelihood that fish would eat the pellets. Cane toad sympatric fish showed a significant decrease in the likelihood of eating distasteful pellets after just a single exposure whereas naïve fish showed no change over the entire experimental period (Fig. 4A). While there was no difference between rainbowfish populations in terms of the number of edible pellets consumed, cane toad sympatric fish were less likely than naïve fish to consume distasteful pellets. Comparison to controls that had never experienced distasteful pellets showed that both populations developed a general aversion of pellets following exposure to distasteful pellets (Fig. 5). These data strongly suggest that natural selection has favoured the evolution of enhanced avoidance learning in cane toad sympatric populations of rainbowfish. Evolutionary responses to cane toads have been reported in terms of both the biochemical response to poison [29] and phenotypic shifts to reduce exposure to toxins [30] in snakes. The data presented here is the first evidence that long term exposure to a toxic invasive species can also result in the evolution of cognitive responses to the threat.

Crimson spotted rainbowfish are capable of learning to avoid predators [38] and habitats associated with predators [39], and can retain information about negative experiences for over a year [35]. Cane toad sympatric fish displayed generalised aversion throughout the study which lasted for several months, indicating that generalised aversion acquired from exposure to cane toad tadpoles had a long-lasting effect on rainbowfish behaviour. These results support those of a previous study which demonstrated that experience with cane toads can produce a generalised aversion of similar prey types [40], [41]. The common planigale, *Planigale maculata,* is a small dasyurid that commonly feeds on frogs. Planigale stopped feeding on frogs for up to 9 days following exposure to cane toads [40]. Similarly, lab experiments examining the effect of exposure to cane toad tadpoles in the northern trout gudgeon, *Mogurnda mogurnda*, showed a general shift away from native tadpoles [41]. Thus it seems likely that the toxic nature of the cane toad, even at the tadpole stage of the life cycle, is severe enough to generate aversion of similar prey types for considerable lengths of time. That a similar response is observed in taxa as diverse as fish and mammals is compelling. While general aversion could protect fish from poisoning by cane toad tadpoles, it could also have a negative fitness effect as well as indirect effects on other aspects of the aquatic ecosystem [41].

Diet changes induced by introduced species can have wide ranging indirect trophic effects [42]. Depending on how important tadpoles are to the diet of predators and the availability of alternative prey, a generalised aversion of tadpoles could have a negative effect on large predatory fish populations. It is obvious that this would lead to increased predation pressure on alternative prey types [41] or a general reduction in predation pressure in the system via a reduction in predator numbers. Ironically, both of these processes can have a positive effect on native tadpole populations [29], [43], [44] and may partly counteract the large number of negative impacts of cane toads on native frog species [27], [45], [46].

To summarise, cane toad sympatric fish showed reduced attacks on both cane toad tadpoles and native tadpoles. Experiment two, suggested that perhaps both learning and evolutionary responses to cane toads were responsible for the differences between the two populations. Nevertheless, both populations were clearly capable of avoidance learning. The introduction of the novel prey type in experiment three further highlighted differences between the populations. Only the cane toad sympatric fish showed a significant reduction in the number of attacks on distasteful prey items and were also significantly less likely to consume them. This result clearly highlights evolutionary shifts in the ability of these fish to learn to recognise and subsequently avoid novel, noxious prey items. Given that the toad exposed population used in this study has been sympatric with cane toads for roughly 60 years [17], one would expect that should be sufficient time for a native species to develop an evolutionary response to an invader [47], [48].
